# On the Mysterious Propulsion of *Synechococcus*

**DOI:** 10.1371/journal.pone.0036081

**Published:** 2012-05-02

**Authors:** Kurt Ehlers, George Oster

**Affiliations:** 1 Mathematics Department, Truckee Meadows Community College, Reno, Nevada, United States of America; 2 Department of Atmospheric Sciences, Desert Research Institute, Nevada System of Higher Education, Reno, Nevada, United States of America; 3 Department of Molecular & Cell Biology, and ESPM, University of California, Berkeley, California, United States of America; University of Zurich, Switzerland

## Abstract

We propose a model for the self-propulsion of the marine bacterium *Synechococcus* utilizing a continuous looped helical track analogous to that found in Myxobacteria [1]. In our model cargo-carrying protein motors, driven by proton-motive force, move along a continuous looped helical track. The movement of the cargo creates surface distortions in the form of small amplitude traveling ridges along the S-layer above the helical track. The resulting fluid motion adjacent to the helical ribbon provides the propulsive thrust. A variation on the helical rotor model of [1] allows the motors to be anchored to the peptidoglycan layer, where they drive rotation of the track creating traveling helical waves along the S-layer. We derive expressions relating the swimming speed to the amplitude, wavelength, and velocity of the surface waves induced by the helical rotor, and show that they fall in reasonable ranges to explain the velocity and rotation rate of swimming *Synechococcus*.

## Introduction

The swimming of the marine cyanobacterium *Synechococcus* has been a longstanding puzzle. *Synechococcus* is ubiquitous in the euphotic zone of the worlds oceans making it a major primary producer. Approximately one third of the open ocean isolates are motile. It moves through seawater at speeds of 5 to 25 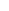
m/s while rotating about its long axis at about 1 Hz [2], [3]. It accomplishes this despite the complete absence of any observable motile apparatus such as flagella. Mechanisms for self-propulsion such as self-electrophoresis and the expulsion of a Newtonian fluid have been ruled out on physical grounds, [4] and [5], leaving the cell surface as the likely location for the generation of thrust. A traveling surface wave mechanism was proposed in the mid 1990's ([6], [7]), but heretofore no mechanism for the generation of the waves has been found. It was shown that a wave with amplitude 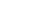, just under the resolution limit of light microscopy, with wave speed 160 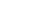 traveling along the cell surface can propel the cell at observed velocities. While this wave speed may seem surprisingly high, we note that even if the entire cell surface were to flow along the cell body, being created at one end and absorbed at the other in a mechanism known as tread milling [8], the outer membrane would still need to move at nearly 40 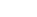 to propel the cell at 25 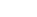. Any mechanism involving cyclic deformations of the cell surface would require much higher surface velocities.

**Figure 1:**
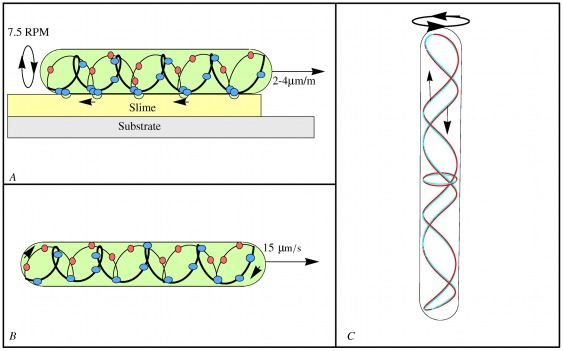
Cytoskeletal helical rotors. (A) Myxococcus xanthus gliding according to Nan, *et al.* [1]. Cargo carrying motors run along the helical rotor track carrying protein “cargo”. The high drag cargo (blue) forms traffic jams on the ventral side creating surface deformations on the surface of the cell. These ridges travel down the cell as the helical structure rotates propelling the cell. The large cargo is deposited at the trailing pole and exchanged for small cargo (red) that creates little drag. (B) *Synechococcus* swimming. Elements of motor cargo (blue and red dots) move along a continuous looped helical track creating distortions along the S-layer. Cargo is represented by blue dots moving from front (right) to back while small cargo elements move from the back (left) to front. (C) A freeze frame of the red/cyan anaglyph movie video S1. The rotor consists of a right-handed helix and a left-handed helix joined at the ends. If the rotor rotates in the counter-clockwise direction, as viewed from above, the right-handed helix causes surface deformations traveling from the top to the bottom of the cell; the left-handed helix causes deformations that travel from the bottom to the top.

A clue to *Synechococcus's* propulsion comes from a bacterium that does not swim, but glides on surfaces. Recent work on the gliding of individual cells of the soil bacterium *Myxococcus xanthus* showed that its motion is associated with rotation of a continuous looped helical track spanning the entire length of the organism [1], see also [9]. The cell reverses its direction periodically in synchrony with the helix reversing its rotation direction. The helix rotation is driven by a transmembrane proton motive force (PMF), and the track is likely composed of actin-like MreB cytoskeletal filaments. The rotating helix was found to interact with MotAB homologues, the stators of the bacterial flagellar motor. The authors proposed a mechanochemical model in which PMF-driven motors, similar to bacterial flagella stator complexes, run along an endless looped helical track, driving rotation of the track. A layer of high viscosity slime causes “traffic jams” in the AgmU associated proteins to form on the ventral side of the organism creating surface deformations. These deformations pass down the cell as the helix rotates creating pressure waves in the slime thereby pushing the cell forward at speeds of several body lengths per minute, as depicted in figure 1A. As MreB and MotA/MotB homologues are common across a wide variety of bacterial species, Nan, *et al.* speculated that a similar mechanism could be responsible for *Synechococcus* motility. This speculation was buttressed by the observation that treating *Synechococcus* with a chemical (A22) that halts MreB polymerization halted swimming within two minutes. *Synechococcus*, however, swims much faster than myxobacteria glide, so the question arises whether the same mechanism could operate over such a wide velocity range. Here we propose a concrete model based on the helical rotor mechanism that can explain most of the swimming characteristics of *Synechococcus*. The purpose of the current work is to demonstrate that the helical rotor demonstrated by Nan, et al. can be scaled up to propel *Synechococcus* thereby opening a new avenue in the search for the machinery behind its propulsion.

Electron microscopy studies of two swimming strains of marine *Synechococcus* reveal that they both possesses crystalline S-layers [10], [1]. It was reported in [12] that a 130-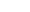 cell surface glycoprotein, SwmA, localized on the cell surface is required for swimming. Cells lacking the gene expressing SwmA do not possess an S-layer [11]. Cells not expressing SwmA are nonmotile, yet when fortuitously attached to a slide, were still observed to rotate at 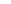
1 Hz about their point of attachment, indicating that the S-layer is required for translation but not rotation. A second surface protein, SwmB, is secreted from the cell surface and is also necessary for swimming, but its role is unknown [13]. Thus while surface proteins appear to be necessary for swimming, their precise role remains unclear. The experiments, however, do strongly implicate the S-layer as an essential component of the swimming mechanism.

In this paper we formulate and analyze a theoretical model for swimming in an unbounded fluid using the machinery of *Myxococcus xanthus*. The basic assumption of our model is the existence of a continuous looped helical rotor analogous to that found in *M. xanthus*, figure 1B. The rotor transmits traveling helical waves to the S-layer which, in turn creates a flow in the surrounding fluid leading to both translation and rotation. The S-layer which is comprised of elongated subunits plays a critical role in our model by providing an asymmetry to the fluid flow and amplifying the height of the waves, greatly increasing the thrust.

**Figure 2:**
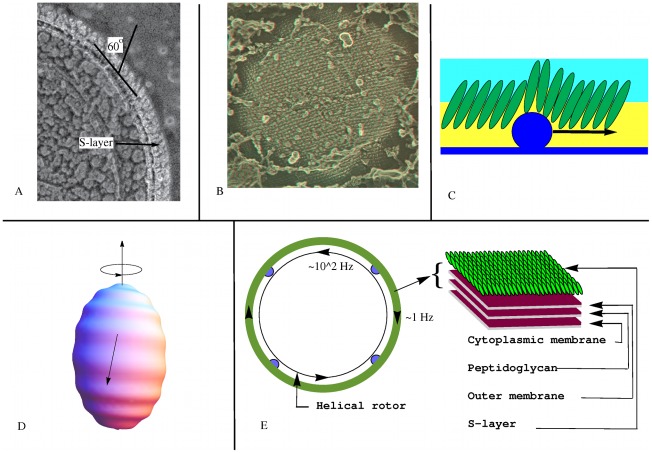
Model 1: Fixed-Motor model for *Synechococcus*. TEM images courtesy of John Heuser. (A) A cross section of the cell wall of *Synechococcus* strain WH8102 showing the elongated subunits of the S-layer inclined at 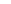
60 degrees. (B) A stereo TEM showing the paracrystalline lattice structure of the SwmA protein S-layer. (C) Amplification of the wave height along the S-layer. The helical rotor, or an element of motor cargo, moving against the grain tilts and displaces the subunits of the S-layer amplifying the surface deformation. The motors (not shown) driving the motion of the helical rotor are attached to the peptidoglycan layer. (D) The motors drive the helical structure to rotate beneath the cell surface in the counter-clockwise direction (as viewed from above). This creates a traveling helical wave passing from the top to bottom, and the cell surface counter-rotates in the clockwise direction. (E) Cross section of Model 1: Elements of motor cargo (blue dots) anchored to the peptidoglycan drive rotation of the helical rotor relative to the rest of the cell, including the cell surface S-Layer. The resulting helical wave causes the cell to rotate at 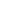
1 Hz relative to the surrounding fluid.

The simplest model for the generation of surface waves involves stationary motors anchored to the peptidoglycan layer. Rotation of the helical rotor transmits helical ridges directly to the cell surface, figure 1C. Alternatively, the motors could move along the helical rotor carrying elements of ‘cargo’ as in *M. Xanthus* [1], figure 1B. In this model the helical rotor counter-rotates with respect to the cell body. Deformations along the S-layer are created by the elements of cargo, figure 2C. The S-layer plays several roles in this model. First, it amplifies the deformation as in the stationary motor model. Its second role is to cause the deformations created by the cargo to be expressed as traveling ridges along the cell surface. Since the motors run in both directions along the cell there must be an asymmetry so that unidirectional thrust is created. In [1] this was accomplished by the exchange of small and large cargo at the ends of the cell. The asymmetry could also occur as a result of the geometry of the S-layer whose component parts appear in electron micrographs as elongated subunits making a tilt of approximately 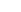 with respect to the cell wall when viewed in cross section. A deformation, created by the rotating helix or moving motor cargo, moving against the grain of the tilted subunits would make large transverse waves while those moving with the grain would make smaller transverse waves.

Our analysis is based on low Reynolds number hydrodynamics. Models of self-propulsion at low Reynolds number by small amplitude surface waves have a rich history in the literature. The seminal paper was written by G. I. Taylor in 1951 who solved the swimming problem for an infinite waving sheet [14]. This model has since been generalized to other geometries including spheres [15], [16], [17], cylinders [17], [18], and ellipsoids [19]. These models were initially developed in an effort to understand flagellar and ciliary propulsion in prokaryotes. The locus of the ciliary tips were assumed to effectively form an envelope allowing the organism to be modeled as a squirming sphere [20]. Here the ciliary tips are replaced with the tips of the subunits comprising the S-layer. A general method for the analysis of swimming by small amplitude waves was derived in [7]. Therefore, much of what we need to test the viability of our models is available in the literature, and we need only adapt these earlier models to the geometry of our models for *Synechococcus* propulsion. Once more detailed information emerges it would be interesting to refine our models using computational techniques such as the immersed boundary method [21] or the computational model recently developed in [22].

## Results

Here we describe and analyze two possible models for the self-propulsion of *Synechococcus* utilizing a continuous looped helical rotor. In the rotating helix model, motors anchored to the peptidoglycan layer drive rotation of the rotor creating helical waves along the cell surface. In the traveling cargo model elements of motor cargo move along the the helical track creating deformations along the cell surface.

Whether the surface deformations necessary for propulsion are created by motor cargo moving along the helical rotor as with *M. Xanthus* or directly by rotation of the rotor itself, the S-layer plays two important roles: it creates an asymmetry in the deformations and it amplifies the distortions. These are accomplished through the geometrical arrangement of the subunits within the S-layer. Electron micrographs of its cross section indicates that it is comprised of elongated protein subunits tilted at an angle of 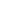 with respect to the cell surface; see figure 2A. As viewed from above, the subunits are arranged in a rhomboidal crystalline lattice (Figure S1). It is the tilt of the subunits that leads to the asymmetry in the cell surface deformations that leads to unidirectional motility.

The helical rotor consists of a left handed helix and a right handed helix connected to each other at the ends, figure 1C, (Video S1). Whether deformations of the cell surface are created by the rotating helix itself as in the rotating helix model or by elements of cargo as in the traveling cargo model, deformations travel from the front of the cell to the rear and vice versa. Without some mechanism creating an asymmetry the resulting fluid flows would cancel. In M. Xanthus exchange of low drag and high drag cargo at the poles creates the asymmetry. In our model of *Synechococcus* it is the tilt of the subunits comprising the S-layer that creates the asymmetry. A section of helix or element of motor cargo moving against the tilt of the subunits causes them to rotate on end creating a relatively large transversal wave while a section of helix or element of motor cargo moving with the grain creates negligible transverse waves and possibly longitudinal waves. Remarkably, transverse and longitudinal waves propel the surrounding fluid in opposite directions [23]. It is therefore possible that deformations running in both directions contribute to the forward propulsion of the cell.

### Rotating helix model

We assume that the helical track rotates creating a small amplitude, high frequency helical wave along the S-layer, figures 2D and 2E. This could be realized either by motors running along the track, as in [1], or by motors anchored to the peptidoglycan layer driving the helical track. If the cell surface assumes a helical pattern due to the interior helix, the helix rotation would be reflected in waves on the surface providing both axial thrust and rotational torque. To test the viability of this model we estimate the pitch and rotational frequency of the helical rotor necessary to propel the cell with observed translational and rotational speeds.

We begin by assuming a constant velocity field on the surface of a prolate spheroid with aspect ratio 2∶1. By assuming a constant velocity field 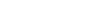, where 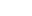 are spherical coordinates with 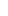 being the azimuthal angle, we determined the constants 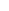 (leading to translational velocity) and 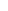 (leading to rotational velocity) such that the cell swims at 15–25 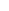
m/s with a rotational speed of 1 Hz. Applying formulas for the translational and rotational speed of a prolate spheroid associated with a constant velocity field on the cell surface derived in [7] we find that 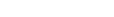 and 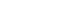. The helical rotor should have between 5 and 10 turns between the ends of the cell.

To estimate the frequency of the rotating helical rotor we appeal to the squirming sphere model of [15] and [16]. This model provides the swimming velocity associated with an axially symmetric traveling wave expanded in a basis of Legendre functions. By changing the basis to a Fourier basis we can estimate the necessary wave speed and from this the rotational frequency of the helical rotor. The required frequency will be somewhat less for an elongated cell. On the other hand, by assuming an axially symmetric wave, we have neglected the relatively small rotational component of the wave.

Assume a traveling wave on a sphere of radius 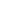 given by
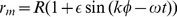
(1)where 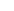 is the radius of a material point on the deformed sphere. Fix a wave number 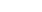 corresponding to 8 cycles between the north and south poles of the sphere. The wavelength is 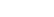 and the wave speed is 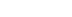. By expanding equation 1 in terms of Legendre polynomials and applying the result of [15] and [16] we obtain
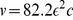
(2)for the velocity of propulsion. The coefficient 82.2 depends on the wave number 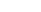. See the Methods section for a derivation of this formula and its generalization to other wave numbers.

To determine the rotation frequency of the helical rotor we match the wave speed necessary to propel the cell at the observed speed to the speed of a wave generated by the rotating helix. For example, assume the helix has 8 turns, and take 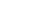
m and a velocity of propulsion of 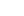
m/s. The wavelength is then 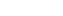
m. If the wave amplitude is 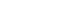
m, the required wave speed is 456 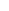
m/s. To generate a traveling wave with this wave speed the rotor must spin with frequency of 1169 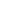. For an amplitude of 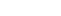
m the wave speed is 73 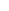
m/s and required frequency of the rotor is 186 Hz. The frequency could be somewhat less if we allow for both transversal and longitudinal waves but would still be on the order of 100 Hz.

By comparison, the flagellar motor of E-coli, a large membrane embedded structure, can rotate at speeds up to 300 Hz (at zero torque) [24]. In the low speed regime (0–200 Hz) the torque-speed curve of the E-coli flagellar motor is approximately constant (2.7–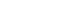
dyn cm) after which it decreases linearly reaching zero at about 350 Hz. The flagellar motor of certain marine bacteria have been observed to rotate at more than 1 kHz [25].

### Traveling Cargo Model

**Figure 3:**
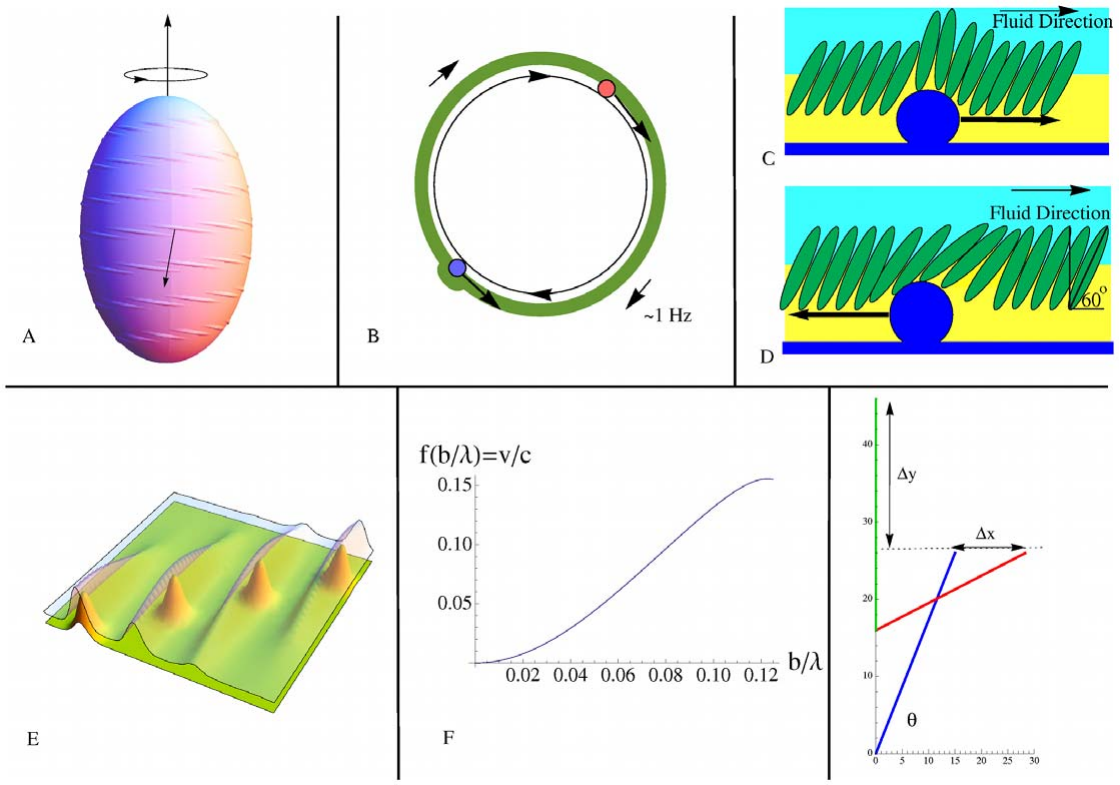
Model 2: Traveling motor model. (A) Deformations created by motor cargo traveling along the helical rotor are expressed as traveling ridges due to sterical coupling of adjacent subunits in the S-layer. (B) Cross section of Model 2: Motor cargo (blue balls) move along the helical rotor (black circle) and are not anchored to the peptidoglycan layer as in Model 1. Motor cargo moving with the grain of the S-layer creates a large deformation while motor cargo moving with the grain creates a small deformation and possibly a compression/expansion wave. Due to the higher drag of cargo moving with the grain the helical rotor is driven in the opposite direction. (C) Motor cargo moving against the grain (to the right in the figure) causes a transversal deformation along the cell surface driving fluid in the direction of the wave. (D) Because of the tilt of the S-layer proteins, an element of motor cargo moving with the grain (to the left in the figure) causes a local expansion of the membrane, making a wave traveling in the direction opposite of the transversal waves shown in C. Although traveling in opposite directions, both waves create fluid flow in the same direction: left to right. (E) Coupling between subunits in rows of the S-layer cause bumps formed by elements of cargo to create ridges in the S-layer. The elements of cargo follow a diagonal line from the bottom left corner to the top right corner while the wave fronts move from left to right. (F) The 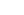 order Taylor approximation to the ratio of the steady fluid velocity 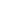 to the speed 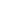 of a transversal wave along an infinite sheet. (G) Here a 16 nm vertical displacement created of a 30 nm subunit at an angle of 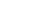 caused by motor cargo moving against the grain creates a transversal wave with height of 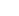 = 20 nm when it rocks on end. If instead the subunit rotates in reaction to motor cargo moving with the grain, the maximum longitudinal displacement would be 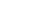 nm.

In this model cargo carrying motor proteins move along a continuous looped helical track in a manner analogous to those found in *M. xanthus* [1], figure 1B. The motor cargo creates surface distortions along the S-layer as shown in figure 3A and B. Since the motor proteins run along the track in both directions an asymmetry in the mechanism must be present to generate unidirectional motion. In *M. xanthus* the asymmetry is created by the exchange of high and low drag cargo at the poles of the cell. This asymmetry is consistent with our model; however, the electron micrograph in figure 2A reveals another possibility. In this cross section, the components comprising the S-layer appear as subunits making an angle of approximately 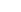 with respect to the cell wall. Elements of motor cargo moving ‘against the grain’ (i.e. against the tilt) could create large transverse surface distortions while motor cargo moving ‘with the grain’ would make smaller transverse distortions (figures 3C and D). The helical track would rotate relative to the cell wall due to the higher drag of motor cargo when running against the grain of the S-layer. The effect of this would be that the helical pattern of the surface distortions would have a larger pitch than does the helical rotor itself.

The ‘rowing’ of a single subunit in the S-layer would produce only a small propulsive force. If the subunits are sterically coupled to their neighbors, a moving motor driving a stroke on one subunit would produce a distributed displacement with decreasing amplitude along a row of subunits, e.g. a moving Gaussian-like wave as shown in figure 3E). The result would be a traveling transverse wave along a ribbon above the helical track, figure 3A. The surrounding fluid would be propelled in the direction of the cargo, thus propelling the cell in the opposite direction, as shown in figure 3C.

This model could explain the observation that cells lacking the S-layer, but otherwise intact, lose the ability to translate but still rotate when attached to a slide [11]. Without the S-layer, the ridges would not form leaving surface distortions in the form of traveling lumps. While the profile of the ridges leading to translation would be greatly diminished, the profile producing rotation would not be significantly changed.

As noted earlier, elements of motor cargo moving with the grain of the S-layer subunits would make small amplitude transverse waves. It is also possible that longitudinal compression-expansion waves could be created due to rotation of the subunit as an element of motor cargo passes by, as shown in figure 3D. While longitudinal waves propel fluid in the direction opposite of the wave direction, compression waves propel the cell in the same direction as the wave [23]. It is possible therefore that elements of motor cargo moving in both directions contribute to thrust in the same direction. As a simple model for the generation of longitudinal waves, let us assume that when an element of motor cargo is pushed with the grain, the interior end of the subunit moves in the direction transverse to the cell wall and the end adjacent to the fluid moves in the longitudinal direction, figure 3G. Simple geometry shows that the amplitude of the resulting longitudinal wave can be significant. For example, if the subunits make an angle of 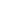 with respect to the horizontal, and the length of the subunits is approximately 30 nm, then the amplitude of the longitudinal wave is about 14 nm. The magnitude of the fluid velocity is the same for longitudinal and transverse waves but in opposite directions [23]. Longitudinal compression waves traveling from the rear of the cell to the front generate flows propelling the cell in the same direction as transverse waves traveling from the front of the cell to the rear. Longitudinal compression waves could effectively double the speed of propulsion associated with transversal waves alone.

Using the formula for the rotational and translational velocity of a spheroid derived in [7] and a generalization of the classical result of GI Taylor for a swimming sheet [14] we derive the formula for a helical ribbon wrapping around the cell 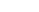 times
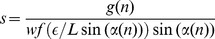
(3)This formula relates the required speed of the motor cargo 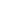 to the average spacing between elements of cargo 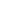, the (average) amplitude of the resulting ridges 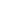, and the width of the helical ribbon 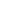, necessary to propel a prolate spheroid with major axis 1 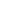 and minor axis 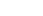 with a translational speed of 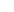 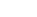 and a rotational speed of 1 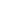. (See the Methods section for details of the derivation.) The distance between successive ridges is approximately 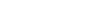. In this formula 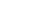 represents the magnitude of a constant boundary velocity field along the ribbon that would lead to the desired propulsive speed, and 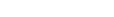 is the ratio of the propulsive velocity of a waving membrane to the wave speed. Values of 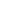 and 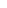 for 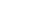 3, and 4 were computed numerically and are given in table 1. Values of 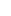 are given in figure 3F.

**Table 1:** Computed values of 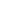 and 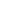, 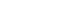.

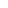	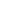	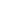	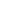	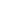	Arclength ( 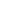 )
2	9.20	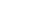	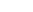	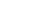	8.59
3	5.54	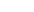	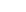	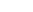	13.74
4	3.98	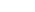	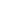	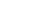	18.81

Formula (3) is based on an approximation that assumes 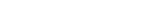. We also assume that the ribbon's width is large in comparison with the amplitude of the ridges. The function 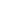 attains a maximum value of 0.155 when 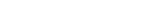. We should note that this is a limitation on the analysis but not on the mechanism itself. Allowance for higher amplitudes or shorter wavelengths would lead somewhat lower values for the required cargo speed 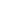.

#### Sample calculation

Suppose, for example, that 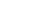 (so that the ribbon wraps around the cell seven times), the average amplitude is 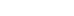 (corresponding to a ridge height of 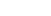), and that the ribbon width is 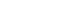. These parameters are depicted in the deformed spheroid in figure 3A. If we take 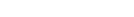 we have 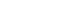. There are approximately 66 elements of motor cargo distributed along the helical track with a spacing of 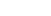. The width of each ridge is approximately 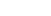 (formula 22). Using formula 3, the necessary speed of the motor cargo is 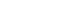. This corresponds to a wave speed of approximately 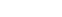 which is consistent with values found in [6] and [7]. Larger values of 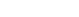 than those allowed by our analysis would lead to smaller required cargo velocities. In our analysis the fluid velocity is 0.155 times the wave velocity. Larger values of 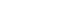 could lead to fluid velocities of perhaps 0.3 times the wave velocity in which case the required motor cargo velocity would be 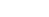. If we allow for the possibility of longitudinal waves being formed by motor cargo moving along the return portion of the helical loop the estimated velocities of the motor cargo could be halved.

## Discussion

We have shown that the same helical rotor mechanism that appears to propel myxobacteria gliding can be adapted to explain the swimming of *Synechococcus*. The list of internal helical structures in rod-shaped bacteria has grown to include most bacterial cytoskeletal proteins. However, it was still startling to find that the back-and-forth gliding of a myxobacterium was driven by a rotating helical loop whose direction of rotation reversed in synchrony with the direction reversals [1]. The helical rotor was powered by a transmembrane ion-motive force via motors that appeared to be related to the ubiquitous bacterial flagellar motor. These motors appeared to run pole-to-pole carrying protein cargos that could be tracked by florescent tagging. In these bacteria gliding is always accompanied by secretion of highly viscous slime. Thus the observation that motors accumulated in periodically spaced ‘traffic jams’ on ventral led naturally to the idea that the aggregations constituted ridges on the ventral surface that traveled from leading to trailing pole as the helical rotor turned. These moving surface ridges drove gliding much as the transverse waves on snail mantles drives their crawling [26]. The authors speculated that this mode of locomotion might be quite general amongst gliding bacteria, and might even be adapted to the swimming of *Synechococcus*. In this work we show mathematically just how this adaptation could work when the bacterium swims through water, whose viscosity is a thousandth that of slime.

The surface layer of proteins (S-layer) is essential to the swimming mechanism of *Synechococcus*. Mutants lacking key surface proteins, SwmA [12], fail to swim, although they continue to rotate. Also, insertional inactivation of the gene encoding SwmB, a giant cell-surface protein arranged in a punctate manner on the cell surface, arrests swimming [13]. By contrast, an S-layer plays no role in myxobacterial gliding, for the slime provides the mechanical coupling between the wave crests and the substrate. In order to swim at the observed velocities in a far less viscous environment, the S-layer proteins are organized and coupled so that the small amplitude of the rotor induced ridges are amplified to provide a sufficient mechanical coupling to the water.

There is little evidence that *Synechococcus* reverses periodically as does *M. xanthus*. Thus the helical rotor in *Synechococcus* may not reverse its rotation direction. This raises the possibility that the rotor motors may be anchored to the periplasm as they are in flagella-driven bacteria such as *E. Coli*. Thus we investigated this possibility as well as the Nan *et al.* model for moving motors. Both could work in *Synechococcus*, and only experiments can distinguish them at this point.

When a swimming *Synechococcus* encounters a barrier to its progress it frequently rotates conically about its leading pole at the same frequency that it did when swimming. This too is explained by the model presented here, for the helical rotor that drives swimming loops at the poles providing a rotating ridge that provides axial torque coupling to the barrier.

Finally, there are observations that beads placed on the surface of *Cytophaga* or of *M. xanthus* move axially along the cell at velocities comparable to their gliding velocities, which differ by an order of magnitude [27], [28]. It is tempting to speculate that these observations are also explained by the helical rotor model. All that is needed is that the beads ‘surf’ on the surface waves generated by the helical rotor. This would require that the beads stick to the cell surface with a weak and nonspecific force, perhaps partially electrostatic. Then the beads would be propelled along the surface, reversing at the poles, leading to counter-propagating beads. These are indeed observed.

While progressive surface waves have been the primary candidate for the self-propulsion of *Synechococcus* since the 90's [6], [7], no mechanism for their generation has been found. We have proposed and analyzed a concrete model for generation of the waves based on a helical rotor that is consistent with the experimental evidence. In particular, the model provides an essential role for the S-layer which is known to be necessary for motility [12].

## Methods

Here we present the mathematical details of our models. *Synechococcus* is a rod shaped bacterium approximately 2 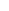
m in length and 1 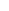
m in diameter. It swims with velocity 15–25 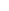
m/s in seawater with viscosity 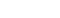 g/cms and density 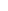 = 1 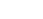. The Reynolds number, 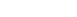, where 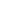 is a characteristic length and 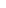 is a characteristic velocity, is on the order of 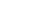 so viscosity dominates over the effects of inertia. Thus the appropriate equations of motion are the Stokes equations
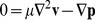
(4)

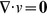
(5)where 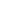 is the fluid velocity exterior to the cell surface 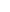 and 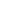 is the pressure [23]. We apply no-slip boundary conditions 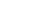 where 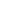 is a vector field representing the instantaneous velocity of the outer membrane of the organism. We assume that the fluid is at rest at infinity. The force and torque associated with 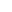 exerted on the surrounding fluid are given by
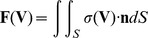
(6)

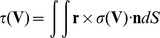
(7)where 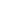 is a position on the cell membrane and 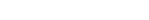 is the stress tensor [29].

The basic principle of low Reynolds number swimming is that, at each instant, the net sum of the forces and torques exerted on the surrounding fluid by a free swimming, neutrally buoyant organism is zero. Let 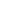 be the vector field on 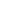 representing the instantaneous velocity of the outer surface of the organism; then at each instant 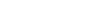. This principal has profound consequences for the self-propulsion strategies available to a microorganism. For example, simple reciprocal motions provide no net motion at low Reynolds number [30]. The time independence of the Stokes equations imply that low Reynolds number flows are reversible. Low Reynolds swimmers must execute non-trivial loops in their shape space to self-propel [17].

For a neutrally buoyant, free swimming organism the vector field 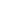 representing the instantaneous velocity of the outer surface can be decomposed into a disturbance vector field (typically not force and torque free) and a vector field 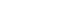 representing the rigid motion of the organism necessary to enforce the zero force and torque condition. In [7] formulas for these translational and rotational velocities associated with an arbitrary boundary vector field were derived using the Lorentz reciprocal theorem. These formulas only require solutions to the Stokes equations for rigid motions of the average shape of the cell, see also [31]. If we specialize to the case of a prolate spheroid 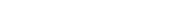 translating along the 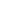 axis with speed 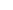 and rotating about the 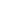-axis with angular speed 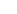 these formulas are
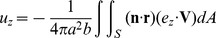
(8)and
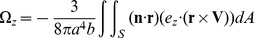
(9)where 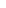 is the position vector on the surface of the spheroid and 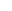 is a unit vector in the 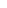 direction. These formulas allow the translational and rotational velocity associated with longitudinal compression waves to be computed directly without further fluid mechanics. In the present case we are interested in motions created by transverse waves, and this requires knowledge of the fluid velocity near the cell boundary in order to compute derivatives in transversal directions.

### 0.1 Fixed motor model

Here we assume that the helical rotor rotates creating a high frequency, small amplitude helical wave along the outer surface. Mathematically, we model *Synechococcus* as a prolate spheroid that propels itself using a traveling helical wave passing from the front of the cell to the rear.

#### Pitch of the helical rotor

Using equations (8) and (9) we can determine the constant velocity field on the surface of a prolate spheroid cell that would lead to the observed combination of rotational and translational velocities. From this constant velocity field we can estimate the angle the helical wave should make with respect to a line of longitude along the cell and the number of times the crest of a helical wave would wrap around the cell. To this end, consider a constant velocity field 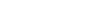 where 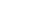 are spherical coordinates with 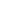 being the azimuthal angle. *Synechococcus* is approximately 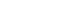 in length and 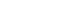 in diameter and swims with velocity 10–20 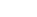 while rotating about its long axis at about 1 Hz. With these parameters 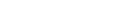 and 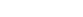. The velocity field makes an angle between 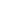 (for 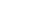) and 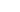 (for 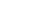) measured with respect to a line of longitude along the cell. The crest of a helical wave generating this velocity field would wrap around the cell between 5 and 10 times.

#### Frequency of the helical rotor

To estimate the rotational frequency of the helix let us consider the simpler case of a axially symmetric waves on a sphere where explicit solutions to the swimming problem are available. From these solutions we can approximate the rotational frequency of the helical rotor by matching the resulting wave speed to the wave speed necessary to propel the cell at observed speeds. A helical wave with a small pitch will generate velocity components leading to both rotation and translation but those leading to translation are much larger than those leading to rotation. On the other hand, an elongated cell will require a somewhat smaller frequency since more of the cell body is parallel to the axis of translation.

Here we assume that the wave is transversal to the cell wall so we need a formula for the velocity analogous to that found in [6] and [7] for purely longitudinal traveling waves. Unfortunately, transversal waves are much more difficult to deal with since, unlike the longitudinal wave case full solutions to the Stokes equations with boundary data prescribed on a sphere are required when time averaging the fluid velocities over a swimming stroke, cf. [17]. (For longitudinal deformations, only solutions corresponding to rigid rotations and translations of the cell body are required [7].) Fortunately, a complete solution for the swimming of a sphere in terms of a basis of Legendre functions is available [15], [16], [17]. For our purposes we need only change the basis to a Fourier basis.

Assume a traveling wave on a sphere of radius 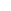 given by
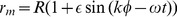
(10)where 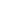 is the radius of a material point on the deformed sphere. The wavelength is 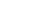 and the wave speed is 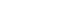. By expanding (10) in terms of Legendre polynomials
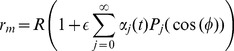
(11)we can apply Blake's result for the velocity of a squirming sphere [16], see also [15], for the velocity of propulsion
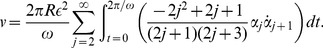
(12)If we fix 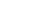 corresponding to 8 cycles between the north and south poles of the sphere the velocity of propulsion is
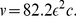
(13)We remark that a truncation of the series is not necessary since only three terms in the expansion of (11) are sequential; only three terms of the series are therefore non-zero. This approximation is valid when 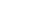. A closed form formula analogous to that for tangential waves found in [6] is only available in the limit of high wave numbers for transverse waves. The velocity of propulsion is asymptotic to
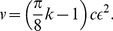
(14)for large wavenumbers 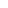. For a small wave number like that considered here, it is best to simply compute the coefficient for the special case.

Take 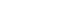 and assume the cell swims with velocity 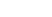. The required wave speed 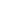 is 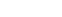 for 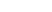 and 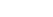 for 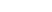. If the wave is generated by a rotating helical rotor (with 8 wraps making the distance between wave crests 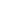), the required frequency of rotation would be 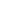 which is 1169 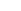 for 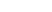 or 186 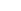 for 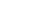. While the helical wave will also have a component leading to rotation of the cell, this calculation gives an indication of the required rotational velocity of the helix.

### 0.2 Traveling cargo model

**Figure 4:**
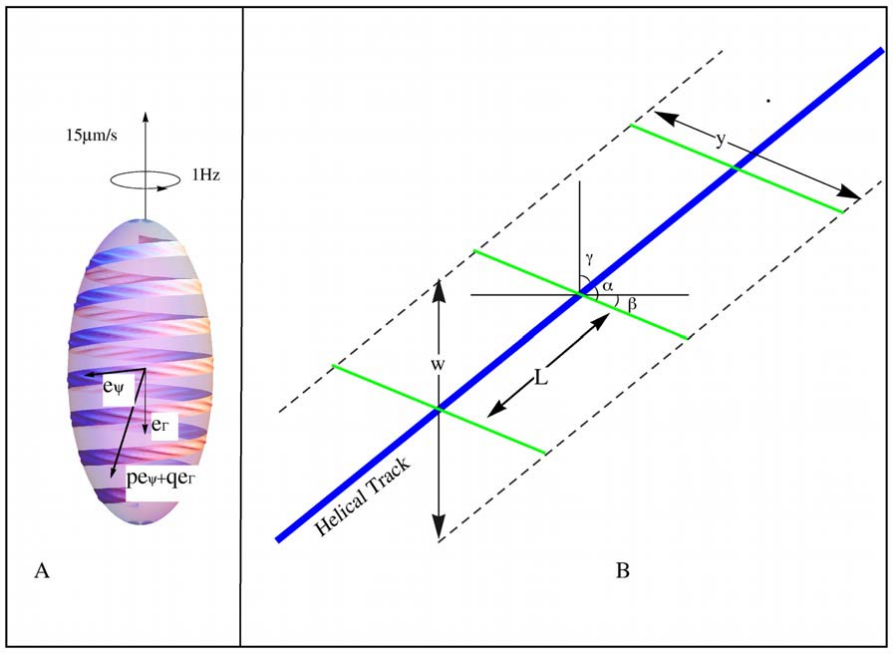
Geometry of the traveling cargo model. (A) Because the proteins of the S-layer are appropriately coupled, motor cargo creates a train of ridges along a ribbon above the helical track of sufficient amplitude to move the fluid media and generate thrust. (B) Geometry of the traveling cargo model: elements of motor cargo spaced at intervals of 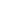 along the helical track (blue line) create transversal ridges represented by the green lines.

In this model, elements of PMF driven motor cargo travel along a helical track creating a train of traveling ridges on the cell surface. The traveling ridges are confined to a helical ribbon on the cell surface, figure 4A.

We derive the formula
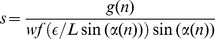
(15)relating the speed of the cargo 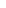 to the number of number of turns of the helix 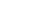, the average spacing between elements of cargo 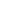, the (average) amplitude of the resulting ridges 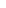, and the width of the helical ribbon (projected onto the long axis) 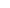, necessary to propel a prolate spheroid with major axis 2 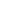
m and minor axis 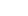 with a translational speed of 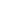 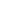
m/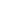 and a rotational speed of 1 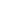. Here all spatial dimensions are in 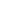
m. The angle between the helical track and the ridges is 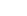, see figure 3B. The distance between successive ridges is approximately 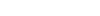. In this formula the quantity 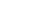 represents the magnitude of a constant boundary velocity field along the ribbon that would lead to the desired propulsive speed, and 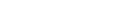 is the ratio of the propulsive velocity of a waving membrane to the wave speed. Values of 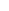 and 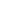 for 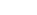 3, and 4 were computed numerically and are given in table 1. Values of 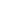, also computed numerically, are given in figure 3F.

The following is an outline of the derivation of formula 15:

1. Determine the components of a velocity field, constant in time, along the helical ribbon representing the surface velocity that, when taken as the boundary condition would lead to a translational velocity of 15 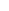
m/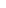 and a rotational velocity of 1 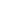.
2. Determine the parameters of a high frequency small amplitude traveling wave that would generate a local velocity matching that found in step 1. We start with Taylor's classical result for the translational velocity for a waving sheet [14]. The geometry of the proposed mechanism leads to relatively small wavelengths so we require more terms in the power series expansion for the fluid velocity than were obtained by Taylor. The necessary terms are obtained using *Mathematica*.
3. Relate the parameters found in step two to the speed and spacing of elements of motor cargo along the helical track.

This method of approximating the swimming velocity, sometimes referred to as the tangent plane approximation [17]. It is explored in more detail in [32] where the method is shown to well approximate the swimming velocity as computed directly for a spherical organism.

#### The velocity field along the propulsive ribbon

Assume for now that the cell can generate a surface velocity field along a helical ribbon on its surface, the velocity field being zero off of the ribbon. The goal of this section is to determine a surface vector field that is constant in time which, taken as a boundary condition on the spheroid, would lead to a translational velocity of 15 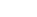 and a rotational velocity of 1 Hz.

We represent the cell as the prolate spheroid 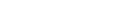 where 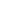, 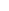, and 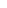 are measured in 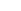. The cell is to swim along the 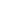-axis. Parameterize the helical ribbon by
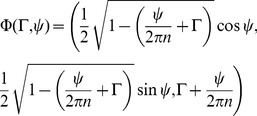
(16)with 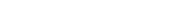 and 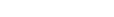, figure 4A. The ribbon wraps around the spheroid 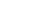 times. The width of the ribbon, when projected onto the 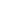-axis, is 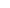. Let 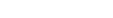 and 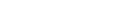 be unit vectors in the 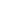 and 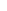 directions. We seek constants 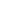 and 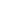 such that the velocity field 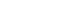 along the ribbon leads to a translational velocity of 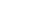 in the 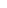-direction and a rotational velocity of 1 Hz about the 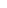-axis.

Setting 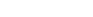, 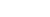, 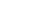, 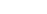, 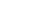, and noting that 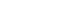 = 0 in formulas 8 and 9, we have
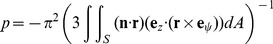
(17)and
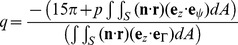
(18)Values of 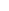 and 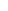 for the special case where the helical ribbon wraps around the spheroid 5 times (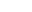) are given in table 2. The third and fifth columns of the table give the values of 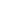 and 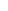 multiplied by the (projected) width of the ribbon 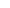. We can see from the data that to good approximation 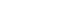 and 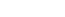.

**Table 2:** Computed values of the velocity components for the case 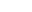.

w	p	pw	q	qw
0.05	106.53	5.31813	19.4864	0.974321
0.09	59.2036	5.31993	10.8314	0.974828
0.13	41.0092	5.3228	7.50486	0.975632
0.17	31.3838	5.32684	5.74555	0.976744
0.21	25.4312	5.33214	4.65801	0.978183
0.25	21.3893	5.33889	3.9199	0.979975
0.29	18.4683	5.34739	3.38678	0.982167

In computing the resultant direction and magnitude we first note that the unit vectors 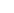 and 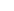 are not orthogonal: the vectors 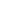 are tangent to meridians but the vectors 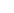 are aligned with the ribbon and are not perpendicular to 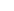. For simplicity, let us use the average angle, 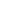, between these vectors. For the special case 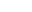, 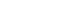. The resultant velocity vector 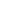 then makes an angle of 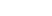 with respect to a line of latitude and has magnitude 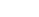, see figure 3B. In general, the magnitude and direction of the resultant depend on 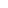:
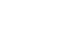

Table 1 gives values for for 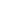, the required angles, as well as the arc length of the center of the helical ribbon for the cases of interest: 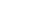, 3 and 4.

The next step is to determine the wavelength, amplitude, and speed of a traveling train of waves along the ribbon that generates this velocity in the adjacent fluid.

#### The traveling wave

Here we determine the parameters of a traveling wave necessary to generate a local fluid velocity of with magnitude 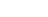 and relate these parameters to the speed and spacing between the elements of cargo creating the wave.

To estimate the local fluid velocity associated with the traveling ridges we appeal to the classical result of G.I. Taylor [14] for the fluid velocity generated by small amplitude traveling waves along an infinite planar sheet. This is a rough estimate since the ribbon is neither planar nor of infinite extent. On the other hand, the oscillatory components of the fluid velocity attenuate as 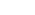 where 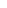 is the normal distance from the sheet [23] and 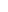 is the wavelength. For our model, the wavelength 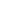 is much smaller than the radius of curvature of the spheroid so we can expect that the local steady fluid velocity is well approximated by assuming that the local geometry is planar.

#### The waving sheet

Here we generalize the classical result of Taylor [14] by obtaining higher order terms. We do this to accommodate the smaller wavelengths (relative to the amplitude) needed for our model. For convenience we maintain the notation used in the original paper; the parameters of the waving sheet will be matched to those of the traveling wave on the spheroid in the next section. Consider an infinite sheet lying in the 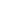-plane in 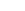-space deforming according to
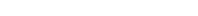
Waves of amplitude 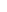 and wavelength 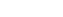 travel to the right with angular frequency 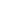. The wave travels with speed 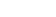. If we assume that the region 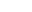 is filled with a viscous fluid, the sheet swims to the left. The speed of propulsion 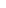 relative to the wave speed 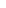 is
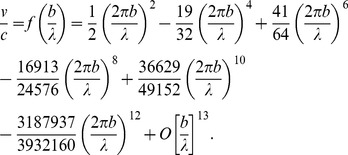
(19)The first two terms were obtained by Taylor [14]. The additional terms were obtained by automating Taylor's calculations on *Mathematica* and retaining higher order terms. Representative values are given in figure 3F.

To apply this analysis to our model we need a sense of how large 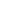 can be without serious error. We follow Taylor's suggestion that the last term be a quarter as large as the sum of the previous terms. For the given expansion this occurs when
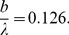
(20)Thus the wave length should be at least 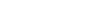 times the amplitude for the approximation to be valid. The analysis can, in principal, be carried out to obtain terms of higher order In in this case, a lower bound (0.75 times the calculated value) on the maximum velocity of the sheet 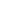 relative to that of the speed of the wave 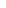 is
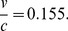
(21)This ratio could be increased to perhaps 0.25 if the traveling wave is allowed to have both longitudinal and transversal components and possibly somewhat higher (but less than 0.5) if 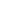 were allowed to exceed the limit of our analysis; see [20].

#### Width of the helical ridges

The length of the ridges 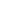 is related to 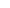 by
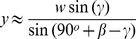
(22)(see figure 4B). For instance, in the case of 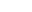 where the ribbon wraps around the spheroid 5 times the length of the ridges 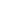 is related to the (projected) width of the ribbon 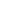 by 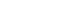.

#### Required speed of the motor cargo

Combining the results from the previous two sections, the required wave speed along the ribbon necessary to propel the cell at 15 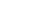 is
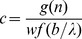
(23)where the values of 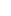 are given in figure 3F and the values of 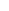 are given in table 1. A traveling wave of with wave velocity 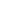 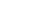, amplitude 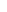 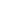, wavelength 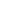 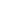, along a helical ribbon of (projected) width 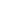 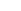 will propel the spheroid with major axis of length 1 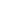 and minor axis of length 0.5 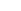 with a translational speed of 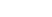 and a rotational speed of 1 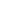.

The last step is to relate this formula to the motion of the cargo along the helical track. We assume that the (average) spacing between elements of motor cargo is 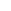 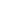 and that they travel with speed 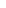 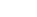 creating traveling ridges of length 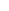 with average amplitude 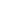. The ridges make an angle of 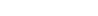 with respect to the helical track, see figure 3B. The length between successive ridges is approximately 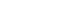 and the wave speed is 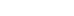. To propel the cell with translational velocity 15 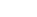 and rotational velocity 1 Hz elements of motor cargo travel with speed given by equation 15.

## Supporting Information

Figure S1 Stereo electron microgram showing the paracrystalline structure of the S-layer.
(TIF)

Video S1 Stereo animation of the helical rotor.
(GIF)
