# Supplementary figures and images for: On the Mysterious Propulsion of Synechococcus
Source: PLoS One. 2012 May 2;7(5):e36081. doi: 10.1371/journal.pone.0036081 (PMC3342319; doi:10.1371/journal.pone.0036081)

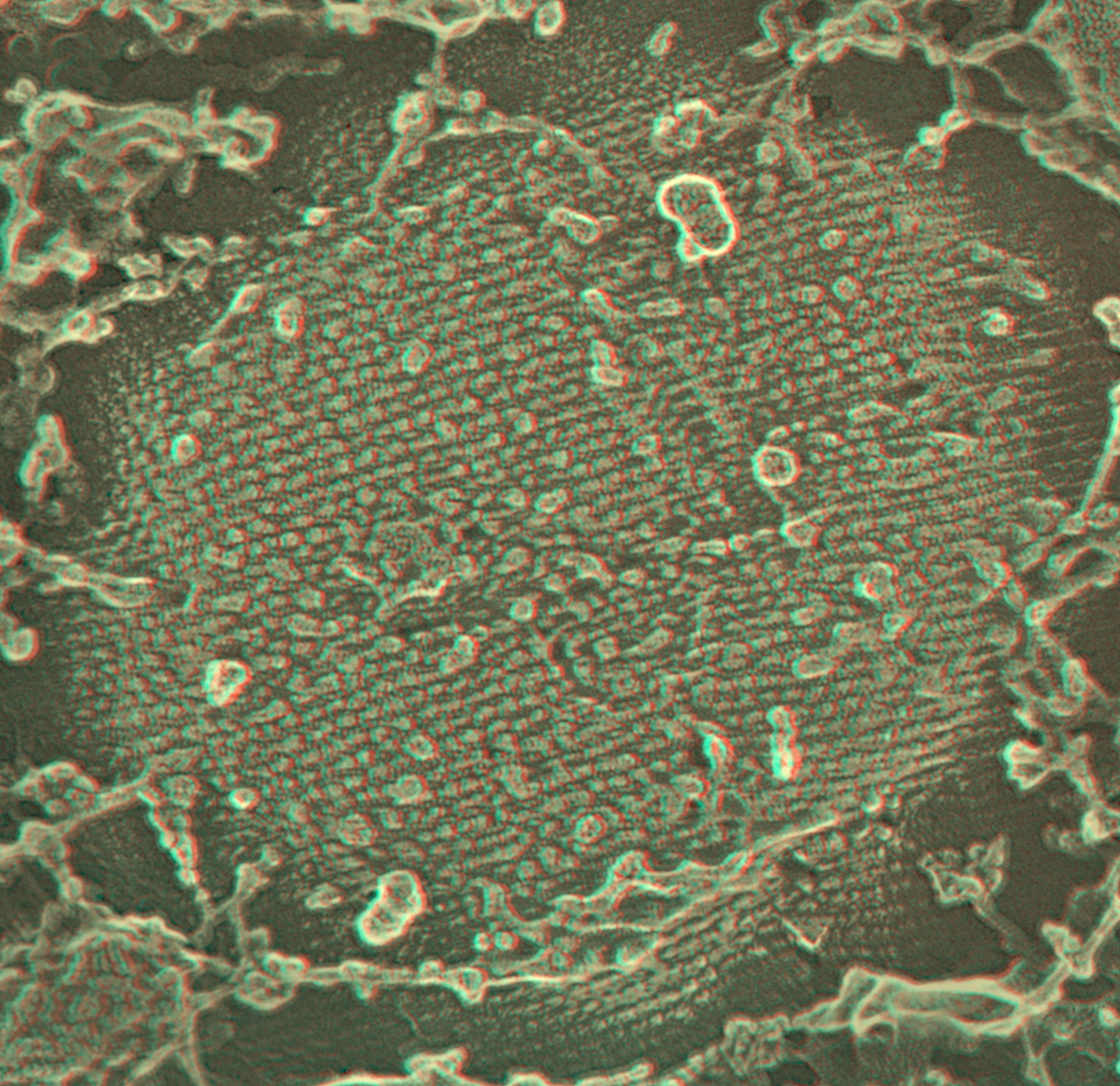

Supplement: Figure S1 — Stereo electron microgram showing the paracrystalline structure of the S-layer. (TIF) [file pone.0036081.s001.tif]

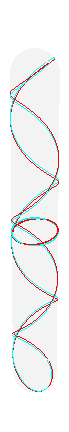

Supplement: Video S1 — Stereo animation of the helical rotor. (GIF) [file pone.0036081.s002.gif]
